# Long-term efficacy of autologous bone marrow mesenchymal stromal cells for treatment of knee osteoarthritis

**DOI:** 10.1186/s12967-021-03160-2

**Published:** 2021-12-11

**Authors:** José María Lamo-Espinosa, Felipe Prósper, Juan F. Blanco, Fermín Sánchez-Guijo, Mercedes Alberca, Verónica García, Margarita González-Vallinas, Javier García-Sancho

**Affiliations:** 1 Orthopedic Surgery and Traumatology Department, Clínica Universidad de Navarra, Pamplona, Spain; 2 Hematology and Cell Therapy Department, Clínica Universidad de Navarra, Pamplona, Spain; 3 Department of Traumatology, Salamanca University Hospital, Salamanca, Spain; 4 Cell Therapy Area, IBSAL- Salamanca University Hospital, University of Salamanca, Salamanca, Spain; 5 University of Valladolid (UVa), Valladolid, Spain; 6 Unidad de Excelencia Instituto de Biología Y Genética Molecular (IBGM), UVa-CSIC, Valladolid, Spain

**Keywords:** Osteoarthritis, Mesenchymal stem cells, Intraarticular injection, Regenerative medicine, Stem cell therapy

## Abstract

Knee osteoarthritis is the most prevalent joint disease and a frequent cause of pain, functional loss and disability. Conventional treatments have demonstrated only modest clinical benefits whereas cell-based therapies have shown encouraging results, but important details, such as dose needed, long-term evolution or number of applications required are scarcely known. Here we have reanalyzed results from two recent pilot trials with autologous bone marrow-derived mesenchymal stromal cells using the Huskisson plot to enhance quantification of efficacy and comparability. We find that cell doses of 10, 40 and 100 million autologous cells per knee provided quite similar healing results and that much of the effect attained 1 year after cell application remained after 2 and 4 years. These results are encouraging because they indicate that, apart from safety and simplicity: (i) the beneficial effect is both significant and sizeable, (ii) it can be achieved with a single injection of cells, and (iii) the effect is perdurable for years.

*Trial registration*: EudraCT 2009-017405-11; NCT02123368. Registered 25 April 2014—Prospectively registered, https://clinicaltrials.gov/ct2/show/NCT02123368?term=02123368&draw=2&rank=1

Osteoarthritis (OA) is the most prevalent joint disease and a frequent cause of pain, functional loss, and disability. It often becomes chronic, and conventional treatments have demonstrated only modest clinical benefits, with no lesion reversal [1]. Cell-based therapies with both autologous and allogeneic mesenchymal stromal cells (MSC) have shown encouraging results in human case reports [2–5]. We have been involved recently in two pilot clinical trials with autologous expanded bone marrow-derived MSC (BM-MSC) where the patients were treated with one single intra-articular injection of cells [2, 3, 5, 6]. The trials showed feasibility, safety, strong indications of potential clinical efficacy and objective improvement of cartilage quality demonstrated by quantitative magnetic resonance imaging (MRI) T2 mapping [2, 3]. In addition, the healing effects seemed to remain for at least 2–4 years after cell treatment [3, 6, 7]. In the present commentary we have performed a more sophisticated analysis using the Huskisson plot [8], which permits quantification of the healing efficacy, in order to compare the different conditions tested (e.g. number of cells) and the endurance of the single shot treatments performed.

The former assay (EudraCT 2009-017405-11; NCT01183728) [2] was performed using a single dose of 40 million autologous BM-MSCs per knee. Feasibility and safety were confirmed and patients exhibited a rapid and progressive improvement of algo-functional indexes (Visual Analog Scale, VAS, and Western Ontario and McMaster Universities Osteoarthritis Index, WOMAC) that amounted 65% to 78% by 1 year, and the amelioration was maintained at the end of the second year [3]. In addition, MRI T2 relaxation measurements demonstrated a significant improvement of cartilage quality.

In the more recent similar trial (Eudra CT 2009-017624-72, NCT02123368) [5, 6] one single dose containing either 10 or 100 million autologous BM-MSCs per knee was used, and compared to controls with no cells. There was no safety issues and the efficacy results were alike with both doses, and at 1 and at 4 years (Fig. 1). However, the baseline values in control and test arms were somewhat inhomogeneous (compare the three red bars in Fig. 1), and this could impair the scrutiny. (Fig. 1). However, the baseline values in control and test arms were somewhat inhomogeneous (compare the three red bars in Fig. 1), and this

**Fig. 1:**
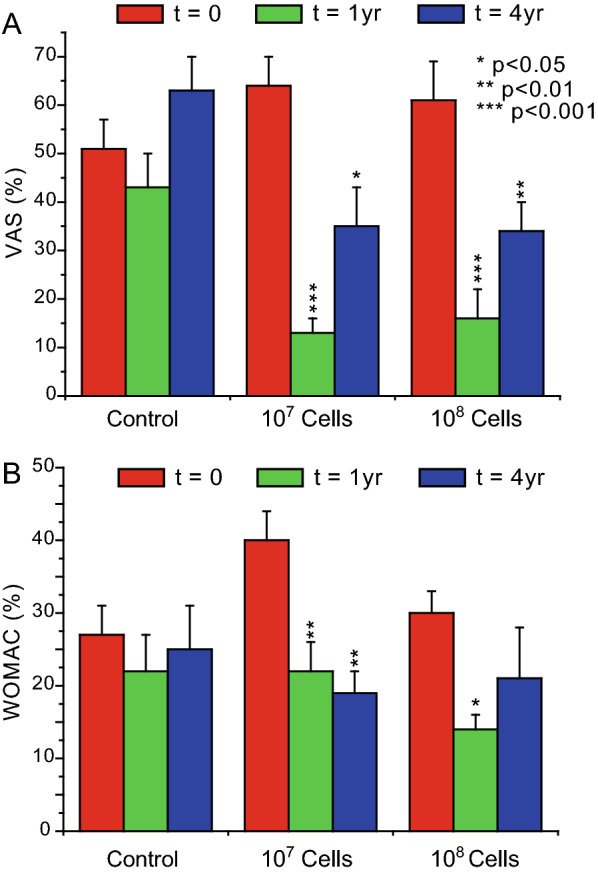
Effects of BM-MSC treatment on pain (**A**, estimated from VAS) and disability (**B**, estimated from WOMAC, general index), both quantified as % of the maximum. Values (mean ± s.e.m. of 8–9 values) before cell treatment (t = 0; in red), 1 year after treatment (t = 1 yr, in green) and 4 years after treatment (t = 4 yr, in blue) are compared in the controls (Gr. 1, hyaluronic acid) and in the cell-treated groups (Gr. 2 and Gr. 3, treated with either 10 or 100 million cells suspended in hyaluronic acid, respectively). Statistical significance was assayed by repeated measurements one-way ANOVA, Bonferroni multiple comparisons; NS, not significant, *p < 0.05; **p < 0.01, ***p < 0.001

A more sophisticated analysis of pain and disability can be performed through Huskisson plots [8]. In this analysis, all the patients are represented (instead of only the mean values, as in Fig. 1), and the improvements to their pain or disability are plotted against their baseline pain or disability score. The Huskisson plot leverages the weights of different baseline values, which otherwise have more or less influence depending on the baseline position within the plot. The result is a regression line where the slope represents a measure of the efficacy of the treatment. A slope of 1 (i.e., the line at 45º in Fig. 2) represents complete (100%) pain relief, the ‘perfect treatment’; conversely, the horizontal line (i.e., slope of 0) represents no healing effect of the treatment at all.

**Fig. 2:**
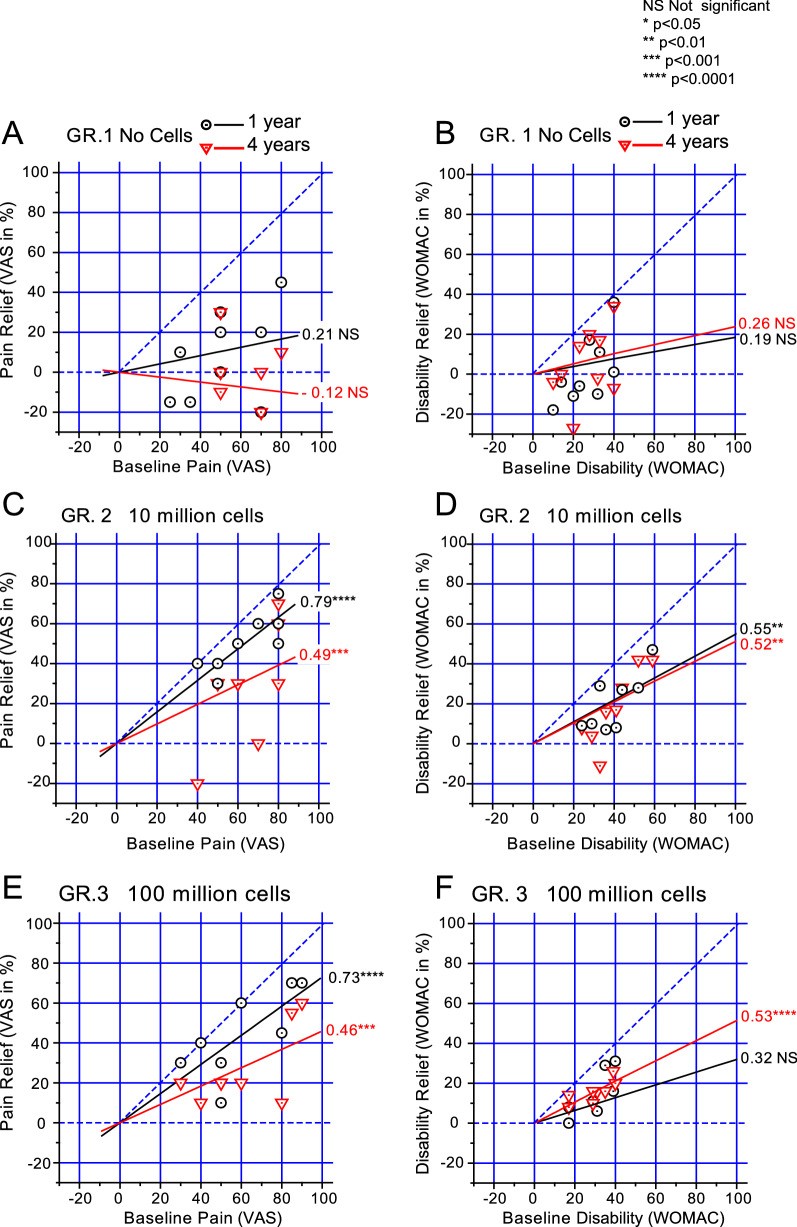
Estimation of the efficacy of the different OA treatments from the Huskisson plot. Data were fitted to a straight line forced to pass through the origin. The slope measures the efficacy of the treatment, and values are given at the right side of the lines. Results from VAS (**A**, **C**, **E**) and WOMAC (**B**, **D**, **F**) in control patients not treated with cells (**A**, **B**), and patients treated with either 10 (**C**, **D**) or 100 million cells (**E**, **F**) are compared. Results 1 and 4 years after cell application are given (black circles and red inverted triangles, respectively). The blue dashed lines represent no effect (horizontal, slope 0) and perfect treatment (45 degrees, slope, 1). Linear regression analysis and statistical significance of the slope (difference from 0) is given. NS, not significant, *p < 0.05; **p < 0.01, ***p < 0.001; ****p < 0.0001

Figure 2 shows the Huskisson plots of the different conditions from the most recent trial [5, 6], using the same scale for all the plots represented. To begin with, the placebo (hyaluronic acid, Fig. 2A and B) had little effect, if any, in all the conditions (Visual Analog Scale, VAS, or Western Ontario and McMaster Universities Osteoarthritis Index, WOMAC, and either after 1 or 4 years of the cell treatment). The slopes of the placebo plots ranged between -0.12 and 0.26, and were not significantly different from zero in all the four cases (see Figure legend for details).

The cell treatments with the 10 and the 100 million cell doses improved the algo-functional indexes, but did not differ very much between them. VAS slopes were 0.79 (Fig. 2C) and 0.73 (Fig. 2E), respectively at 1 year, and 0.49 (Fig. 2C) and 0.46 (Fig. 2E) at 4 years. For WOMAC, slopes were 0.55 and 0.32 at 1 year, and 0.52 and 0.53 at 4 years (Fig. 2D and F). The slopes obtained in the Huskisson plot may also be used for comparison among different trials. It should be remarked that the slopes representing the efficacies of healing of treatments with a dose of 40 million cells was 0.65 to 0.78 after 1 year and 0.71 after 2 years [2, 3], so that the values obtained in the different trials were quite comparable.

In conclusion, the results of the two studies reanalysed and reviewed here demonstrate that, although the presence of the injected BM-MSCs is ephemeral [9], the beneficial effects on knee OA remain for at least 4 years [6]. The healing action seems quite similar for 10, 40 or 100 million cells doses. This unexpected result may arise from saturation of the healing effect and/or from cell damage during transport by oxygen and substrate starvation at the high cell densities used. We have preliminary results that suggest a decrease of cell viability during long storage periods at high density, even at 4 ºC. Long lasting effects of MSC treatments have been attributed to epigenetic actions [9, 10], and this mechanism could explain our results. These observations are of great practical importance as they permit to accomplish cell application with one single cell treatment, which is cheaper and more straightforward than multi-application, and does possible the administration of the cell treatment to a larger number of patients. In this regard, we have adopted this strategy in the design of a new phase III randomized clinical trial in this setting (ARTROCELL trial, with clinicaltrials.gov identifier code NCT05086939) that is currently recruiting patients.

## Data Availability

The datasets used and/or analyzed during the current study are available from the corresponding author on reasonable request.

## Abbreviation

MSCs: Mesenchymal stromal cells
BM-MSCs: Bone marrow-mesenchymal stromal cells
MRI: Magnetic resonance imaging
OA: Osteoarthritis
VAS: Visual analogue scale
WOMAC: Western Ontario and McMaster Universities Osteoarthritis Index
